# Progress in State Medicine

**Published:** 1901-06-08

**Authors:** 


### PROGRESS IN STATE MEDICINE.

**Preventive Measures against the Spread of Measles and Whooping Cough.**—In Great Britain[1] 60 per cent. of the deaths from measles occur in children under two years of age; about 90 per cent. among those under five and about 98 per cent. among those under ten. Probably half the fatal cases are children between the ages of six months and two years. In England and Wales measles attains a maximum at two periods in the year—in June and December. The case mortality of measles varies in different epidemics, in relation to age, previous state of health, and hygienic surroundings. The chief causes of high mortality are overcrowding and insufficient ventilation. The mortality of persons attacked varies greatly with age. It is highest between the ages of six months and two years, and falls rapidly after the fourth year. The death rates [2] per 10,000 population in England and Wales from 1880 to 1889 inclusive are: measles 4·46, whooping cough 4·56; in Ireland: measles 1·95, whooping cough 3·06; Scotland: measles 3·65, whooping cough 6·00.

Bryce [3] discussing how far mandatory measures are of practical value in measles and whooping cough, points out that these diseases require notification under the Ontario Health Act, but that in practice the law is a dead letter. The reasons for this are twofold—firstly the generally mild character of the disease *per se*, secondly the inadequate means at the disposal of the health officers for dealing with epidemics. Statistics show that in England while smallpox and scarlatina have shown an enormous and steady decrease in mortality, comparing the five decades 1851–1895, whooping cough has only shown a decrease of 10 per cent., and measles has remained practically constant, and has a total mortality equal to 50 per cent. of all the other zymotic diseases put together. In spite of this health authorities do not generally recommend, or the public submit, in the case of measles and whooping cough to the measures in general operation regarding smallpox and scarlet fever. With regard to measles the difficulty lies in the fact that the infection is greatest in the earliest stage. Given a single case of measles of school age a localised epidemic of some extent is almost inevitable. Owing to the low mortality of measles among the richer classes, quarantine for from three to six weeks would not be submitted to, and notification would not be followed out even if such were attempted. The poorer classes treat measles with "home remedies" until some complication supervenes. Kenwood's statistics show that even if but 30 per cent. were isolated in hospitals, as much accommodation would be required in a year as for all other contagious diseases together. Bryce recommends that the school authorities should report to the health officer every absentee; immediate investigation of every absentee reported sick by the inspector or truant officer; compulsory notification by the householder of every case occurring in the house; thorough disinfection of the house, articles, books, &c. With regard to the whooping cough, the teachers in schools should be kept well informed of the early symptoms, and instructed to send any child from school who has a suspicious cough, until the true nature of the disease has been ascertained. Notification to the health authorities of the action taken would naturally follow. The isolation by a placard of the children of a household for six weeks would be a hardship and difficult to put in practice, but unless this is carried out the spread of the disease in a district is inevitable.

Probst [4] recommends for measles and whooping cough notification, placarding, domiciliary restraint of the patient and all susceptible children in the family, with official supervision of disinfection. Schools should be closed for a time in any district where either disease threatens to become epidemic, and the school buildings disinfected before they are re-opened.

Measles [5] causes a higher mortality than any of the chief infectious diseases except diarrhœa. Notification, even if not facilitating preventive measures, would furnish a mass of epidemiological data of steadily increasing value.

Notification followed by prompt measures would stop many epidemics. Parents must be educated to realise that measles is a serious disease.

Notification is expensive. In the Croydon Rural District during the years 1891–6 the cost of notification of measles was almost exactly double that of all other notifiable diseases together. In the Urban division of the district the notification of measles cost ½d. per head per annum, and the other diseases about ¼d. per head per annum. On the basis of the whole of the Croydon Rural District, general notification of measles for the whole country would cost £50,000 per annum. The sum is not large if compared with the benefits to be secured from the data collected. Expense is lessened by arranging that only first notification at each house would be paid for.

Theodore Thomson, in a report to the Local Government Board, recommends the compulsory or voluntary notification of measles. The sanitary authority should be informed by the school authorities regarding occurrences of measles. Careful inquiry should be made in each instance of the possible source of infection, with a house-to-house visitation if needful. On the appearance of an epidemic handbills should be distributed to householders pointing out their duty in notifying cases. An officer of the Health Department should visit every infected house and ascertain what steps have been taken to the proper isolation of the sick person. If provision is unsatisfactory the patient should be removed to an isolation hospital. The house should be disinfected, and the members of the household kept under observation for not less than fourteen days after. Sanitary authorities should enlist on their side the school authorities to aid them in excluding from school children suffering from measles, and all members of families in which measles has broken out, until such time as the children may with safety to others be readmitted. The closing of schools should be postponed until the prospect of benefit from other means has well-nigh disappeared. Further methods calculated to check epidemics are precautions against dissemination of measles by means of books sent from public libraries; instruction of the public in the death-dealing effects of measles, and the provision of a sufficient sanitary staff. Sykes, [6] in his report to the Metropolitan Borough of St. Pancras as to the advisability of including measles in the term "dangerous infectious diseases," points out that the Metropolitan Asylums Board has not made, and does not appear to intend to make, any provision for the isolation of measles in hospitals. Without this all measures taken to prevent the spread of infection must prove ineffectual. Compulsory disinfection of infected houses without compulsory notification of all cases would not be likely to check measles epidemics in schools. This disease prevails mostly amongst children under five years, and it creeps into schools mainly through the baby and infant classes. It would materially help in the consideration of means of prevention to weigh the relative effects of school attendance upon "infants" in respect to the amount of education gained and the amount of infection spread. The Sanitary Authorities [7] can institute legal proceedings against any persons contravening the Public Health Act. Also under Section 88 of the Education Code any two members of the Sanitary Authority, acting under the advice of the medical officer of health, can enforce the closure of any elementary day school. The school authorities have the right of appeal, after closing the school, to the Education Department. Sections 81 and 101 of the Code allow the Sanitary Authorities to exclude from school attendance individual children or groups of children when such measures appear necessary to prevent the spread of disease. An extension of legal powers to similarly deal with private, secondary, and Sunday schools is urgently required. School teachers must co-operate with the medical officers of health in supplying him with a list of absentees, where absence is suspected to be caused by infectious disease. Where this has been put in force it has been found to be of the greatest service in preventing spread of infectious diseases. The medical officer of health should be provided with a larger staff of medical inspectors. Owing to expense the poorer classes do not seek medical aid for ailments which appear to them slight, although such ailments are in reality a frequent cause of school epidemics. This can only be remedied by having medical aid available for the working classes gratuitously in every district without any implication of pauperism being involved in securing its advantages. It would involve a State Department of medical aid for free diagnosis, if not for free treatment, in the first instance at least. No parent could then plead that consideration of expense had prevented medical aid being called in, and the sanitary authorities, school boards, and other school authorities would greatly benefit. Davies[8] referring to measles points out that the disease is not notifiable, that its early infectivity and rapid spread almost precluded its control by hospital isolation and disinfection, and that during any wide prevalence extraordinary ample ward accommodation would be required to adequately deal with the epidemic. Much of the immense mortality from this disease might be prevented by careful nursing during the stage of convalescence. Control of school attendance would seem to be the most hopeful method of procedure, and heads of schools can most effectually further this end by giving information of cases and of families known to be infected. This procedure never appears, however, to be wholly effective. In Bristol the measles mortality per 100,000 living was 11. Of the 38 deaths, 36 were of children under 5. Of the 118 deaths from whooping cough, 114 were of children under 5; the mortality in this disease, as in measles, being due to the want of care exercised during the course of the disease, and avoidance of exposure to inclement winds and weather.

[1] System Med. Cliff. Alb., vol. 1. [2] Ibid, vol. ii. [3] B.M.J., Sept. 25, 1899. [4] Ibid. [5] Practitioner, Nov. 1900. [6] B.M.J., March 2, 1901. [7] Practitioner, Feb. 1901. [8] Report Med. Off., Bristol, 1900.

